# Reply to “Bridging experimental biology and clinical practice: lessons from autoinfective larvae of *Strongyloides stercoralis*”

**DOI:** 10.1128/jcm.01435-25

**Published:** 2025-11-13

**Authors:** Huan Zhao, Anson V. Koehler, Cameron Truarn, Damien Bradford, David W. New, Robin B. Gasser, Harsha Sheorey, Richard S. Bradbury

**Affiliations:** 1 School of Public Health and Tropical Medicine, College of Medicine and Dentistry, James Cook University 104560, Townsville, Queensland, Australia; 2 Department of Veterinary Biosciences, Melbourne Veterinary School, Faculty of Science, The University of Melbourne 98487, Parkville, Victoria, Australia; 3 PathWest Laboratory Medicine, QEII Medical Centre 56375 , Nedlands, Western Australia, Australia; 4 Microbiology Department, St. Vincent's Hospital 60078, Fitzroy, Victoria, Australia; Mayo Clinic Minnesota, Rochester, Minnesota, USA

**Keywords:** *Strongyloides*, life cycle, morphology, biology, helminths

## REPLY

We thank the author for their thoughtful commentary on our recent publication, “The fourth-stage autoinfective larva of *Strongyloides stercoralis*: redescription and diagnostic implications” and for highlighting its broader biological and clinical relevance. We appreciate the opportunity to clarify the scope and intent of our work.

Our primary objective was to redescribe autoinfective fourth-stage larvae (L4) recovered from human respiratory specimens, with the aim of supporting laboratory diagnosticians who may encounter these stages in extra-intestinal samples (1). This is particularly relevant in cases of hyperinfection or disseminated strongyloidiasis, where accurate morphological recognition is essential to prevent misidentification, diagnostic confusion, and delays in initiating appropriate treatment (2–4). Accordingly, our discussion was limited to morphological and diagnostic aspects, and we did not explore broader biological implications, as our data did not support such conclusions.

The anatomical site of larval molting remains a subject of ongoing debate (5). Our study did not investigate this question, as our available data did not provide sufficient evidence to determine the location of ecdysis within the human host. Identifying the site of molting is important for a comprehensive understanding of the parasite’s life cycle and biology. We appreciate the hypotheses proposed by the author and thank them for contributing to this important discussion. However, we have deliberately refrained from extrapolating beyond the data presented.

Pulmonary migration is a well-established component of the *S. stercoralis* life cycle (5) and is clinically exemplified by Loeffler’s syndrome, an eosinophilic pneumonitis associated with larval transit through the lungs (6). In uncomplicated chronic strongyloidiasis, only third-stage infective or autoinfective larvae are known to traverse pulmonary tissue (5). Reports of adult worms recovered from the lungs are almost exclusively associated with systemic strongyloidiasis, typically occurring in the context of profound immune dysregulation (7–9). Although clinically significant, pulmonary larval maturation should be interpreted with caution and not assumed to represent the parasite’s typical developmental pathway in immunocompetent hosts.

Historical experimental work by Aikens and Schad (10) using radiolabeled larvae in canine models demonstrated that, although pulmonary passage is central to the parasite’s development, an alternative migratory route exists in which larvae travel directly through visceral tissues to the intestine without entering the lungs. While interspecies variation must be considered, it is reasonable to propose that this alternative pathway, proven in dogs, may also occur in humans (5). These findings emphasize the biological plasticity of *S. stercoralis* and caution against overgeneralizing from any single developmental trajectory.

In conclusion, our study was intended to support diagnostic accuracy by redescribing autoinfective L4 and highlighting the potential for this little-known life cycle stage to complicate laboratory identification and diagnosis. Broader questions regarding the biology of the parasite, including the precise site of molting, remain to be resolved through further research. We are grateful to the author for raising these important issues and echo their call for continued interdisciplinary dialogue. Ongoing collaboration across biological, diagnostic, and clinical domains will be essential to advancing our understanding of *S. stercoralis* and improving outcomes for affected individuals.
